# The Multi-Ethnic Study of Atherosclerosis-Calcium Score Improves Statin Treatment Allocation in Asymptomatic Adults

**DOI:** 10.3389/fcvm.2022.855390

**Published:** 2022-07-15

**Authors:** Gadi Shlomai, Joseph Shemesh, Shlomo Segev, Nira Koren-Morag, Ehud Grossman

**Affiliations:** ^1^Department of Internal Medicine D and Hypertension Unit, Chaim Sheba Medical Center, Ramat Gan, Israel; ^2^The Division of Endocrinology, Diabetes and Metabolism, Chaim Sheba Medical Center, Ramat Gan, Israel; ^3^Sackler Faculty of Medicine, Tel-Aviv University, Tel-Aviv, Israel; ^4^Grace Ballas Cardiac Research Unit, Chaim Sheba Medical Center, Ramat Gan, Israel; ^5^Periodic Examination Center, Chaim Sheba Medical Center, Ramat Gan, Israel; ^6^Department of Epidemiology and Preventive Medicine, Ramat Gan, Israel

**Keywords:** coronary calcium score, cardiovascular events, statin treatment, allocation, prognosis

## Abstract

**Background:**

The current categorization of cardiovascular (CV) risk broadens the indications for statin therapy. Coronary artery calcium (CAC) identifies those who are most likely to benefit from primary prevention with statin therapy. The multi-ethnic study of atherosclerosis-calcium (MESA-C) includes CAC for CV risk stratification.

**Objective:**

We aimed to establish whether the MESA-C score improves allocation to statin treatment in a cohort of asymptomatic adults. We also analyzed patient survival according to their risk score calculation.

**Design:**

A retrospective analysis of asymptomatic adults.

**Participants:**

A total of 632 consecutive subjects free of coronary artery disease (CAD) and/or stroke, mean age 56 ± 7 years, 84% male, underwent clinical evaluations and CAC measurements.

**Main Measures:**

PCE and MESA-C risk scores were calculated for each subject. According to the 10-year risk for CV events, subjects were classified into moderate and high CV risk (≥7.5%) for whom a statin is clearly indicated, or borderline and low CV risk (<7.5%).

**Key Results:**

During mean follow-up of 6.5 ± 3.3 years, 52 subjects experienced their first CV event. Those with a MESA-C risk score < 7.5% had favorable outcomes even when the PCE indicated a risk of ≥ 7.5%. The MESA-C score improved the discrimination of CV risk with the ROC curves C-statistics increasing from 0.653 for the PCE to 0.770 for the MESA-C. Of those, 84% (99/118) with borderline CV risk (5–7.5%) according to the PCE score, were reallocated by the MESA-C score into a higher (≥7.5%) or lower (<5%) CV risk category. Furthermore, subjects with low MESA-C scores had the highest survival rate regardless of the PCE risk, while those with high MESA-C risks had the lowest survival rate regardless of the PCE risk.

**Conclusion:**

In asymptomatic subjects, the MESA-C score improves allocation to statin treatment and CV risk discrimination, while both scores are essential for more precise survival estimations.

## Introduction

The recent American College of Cardiology/American Heart Association (ACC/AHA) guidelines on managing blood cholesterol for primary prevention emphasizes the need for individual cardiovascular (CV) risk stratification (1). According to society guidelines, the categorization of CV risk is mainly based on the pooled cohort equations risk calculator (PCE) (1, 2), which significantly broadens the indication for statin therapy (3, 4). However, long-term statin treatment can expose subjects to unwanted side effects (5, 6) and hence, should be avoided in low-risk subjects. Guidelines suggest using risk modifiers to improve statin therapy allocation for primary prevention (1). Coronary artery calcium (CAC) is among the most powerful prognostic tools for CV events and mortality (7–21). It has been shown that the presence and severity of CAC helps to identify patients who are most likely to benefit from statins for primary prevention (22). Furthermore, subjects with zero CAC have excellent CV prognosis (23–29), and most of them are recategorized into the lower CV risk category (<5%). Consequently, CAC was accepted by both the ACC/AHA (1) and the European Society of Cardiology (6) as a risk modifier for statin treatment.

The multi-ethnic study of atherosclerosis-calcium (MESA-C) score is the first to include CAC, in addition to traditional risk factors, to estimate the 10-year cardiac risk (30). CAC has also been shown to be an independent predictor of cerebrovascular accidents (CVA) in asymptomatic adults (31). The MESA-C score has been validated in the Heinz Nixdorf Recall (HNR) study (32) and the Dallas Heart Study (DHS)(33). Recently, a study on a large retrospective cohort of asymptomatic patients has shown that the MESA-C score and PCE plus CAC showed the best CV risk discrimination among patients with an estimated CV risk of 5–20% and that the addition of CAC also modestly improved risk discrimination among those estimated as low or high CV risk (34).

We aimed to identify the proportion of asymptomatic patients for which using the MESA-C score will result in reallocation to statin treatment by recategorization to a higher or lower risk category.

## Materials and Methods

### Study Population

A total of 1,850 subject underwent annual screening in our clinic between January 2001 and January 2002. We included men age > 40 and women age > 50 years who were CAD and/or stroke free, consented to perform a screening cardiac CT for CAC, and had complete baseline clinical and laboratory data.

All CT scans were performed on a spiral multi-slice CT without contrast injection in a single center and analyzed by an experienced physician (JS). The scanning protocol and CAC measurements were done according to the Agatston method (35).

Blood pressure (BP) was measured in a seated position after 3 min of rest. Hypertension was defined when two separate BP readings were ≥ 140 mm Hg for systolic BP and/or ≥ 90 mm Hg for diastolic BP, a history of hypertension was reported, or the subject took antihypertensive medications.

Diabetes mellitus (DM) was defined as ≥ 7.0 mmol/L (126 mg/dl) fasting plasma glucose on two separate readings, a history of DM was reported, or the subject takes insulin or oral hypoglycemic medications. Hypercholesterolemia was defined when measured total cholesterol was > 6.48 nmol/L (250 mg/dl) or when the patient reported taking cholesterol-lowering medications. Smoking status was derived from the questionnaire, and participants were divided into current smokers and non-smokers.

The estimated glomerular filtration rate (eGFR) was calculated according to the chronic kidney disease epidemiology collaboration equation.

The PCE score estimating the 10-year CV risk (2) and the MESA-C score estimating the 10-year cardiac risk (36) were calculated for each subject. Parameters included in the MESA-C risk calculator were: age, gender, CAC, ethnicity, presence of DM, current smoking, family history of coronary heart disease (CHD), total cholesterol, HDL cholesterol, systolic blood pressure, and the use of anti-hypertensive or cholesterol medications. The PCE score includes the same parameters but does not include CAC, a family history of CHD, and cholesterol medication.

Subjects were followed until the end of 2012. Subjects who were lost to follow-up were contacted, and the coronary endpoints were assessed by a telephone interview. The endpoint was delineated as the first CV event: cerebrovascular accident/transient ischemic attack, acute myocardial infarction, hospitalization for unstable angina, or coronary catheterization that resulted in angioplasty or coronary artery bypass surgery.

### Statistical Methods

Data were analyzed according to the risk categories for statin eligibility as defined by the PCE recommendations as follows: low risk with < 5% 10-year risk for whom a statin is not indicated, borderline risk of 5–7.5% for whom a statin is not definitely indicated and ≥ 7.5% for whom statins are indicated. We further analyzed the data according to two risk groups borderline and low < 7.5% or moderate and high risk of ≥ 7.5%.

To estimate the recategorization value of the MESA-C risk score, we further divided our subjects into four subgroups: those who had both methods a risk < 7.5%, those who had by both methods risk of ≥ 7.5%, those who had a PCE risk of ≥ 7.5% but MESA-C risk of < 7.5% and those who had PCE risk < 7.5% but a MESA score ≥ 7.5%.

Data were presented as mean and standard deviation for continuous variables and as frequency and percentage for categorical variables. Chi-square tests and one-way ANOVA tests were used to compare the groups by baseline characteristics and major risk factors. Event rates and CV risks were calculated for each group.

Cox proportional hazards regression models were constructed to evaluate the hazard ratios (HR) and 95% confidence intervals for CV events in groups classified according to MESA-C and PCE scores. Time to CV event was calculated as the time from screening to CV event, or time from screening to end of follow-up. Multivariate analysis using a Cox regression model was performed with conventional risk factors, selected for the final model on the basis of an association with a CV event in the univariate analysis or findings of significant associations in previous studies. Risk scores were introduced to the model as four categories based on MESA-C and PCE scores. Groups risk differences were demonstrated by the cumulative event-free curves generated from the Cox regression model. The model’s goodness of fit was evaluated by the C index.

Receiver operating characteristic (ROC) curves were used to compare the predictive ability of MESA-C and PCE scores. The ROC curve plots the one minus specificity vs. the sensitivity of each possible cut-point risk on the continuum of the predictor variable. An area under the curve above 0.50 shows the ability of the model to predict cardiovascular events. Data were analyzed with SPSS software version 25.0. The significance levels were set at 0.05.

The research protocol was approved by the local institutional review board and complies with the Declaration of Helsinki.

## Results

A total of 1,850 subjects underwent annual screening as noted above. Of them, 745 asymptomatic consecutive men age > 40 and women age > 50 years who were CAD and/or stroke free consented to perform a screening cardiac CT for CAC. A total of 113 subjects were subsequently excluded from the final cohort due to missing or incomplete data. Thus, our final cohort consisted of 632 participants (84% male) with a mean age of 56 ± 7 years (Table 1). Subjects with low risk by PCE (<7.5%) and high risk by MESA-C (≥7.5%) had a higher CAC and a higher prevalence of CAC above zero than subjects with high PCE scores (≥7.5%) and low MESA-C scores (<7.5%) (Table 1). All other basic characteristics are outlined in Table 1.

**TABLE 1:** Baseline characteristics by PCE and MESA risk groups.

	*Total N* = 632	PCE risk ≤ 7.5% *N* = 347		PCE risk > 7.5% *N* = 285		
		MESA-C risk ≤ 7.5% *N* = 300	MESA-C risk > 7.5% *N* = 47	MESA-C risk ≤ 7.5% *N* = 134	MESA-C risk > 7.5% *N* = 151	
PCE score (units)	8.95 ± 7.6	3.97 ± 1.7	5.16 ± 1.5	12.5 ± 5.2	16.9 ± 9.3	<0.001
MESA-C score (units)	6.79 ± 6.5	2.86 ± 1.6	11.9 ± 3.9	4.1 ± 1.6	15.4 ± 6.8	<0.001
Age (years)	56 ± 7	53 ± 4.6	54 ± 4.0	59 ± 6.6	61 ± 6.3	<0.001
Male gender, *n* (%)	532 (84)	220 (73)	42 (89)	123 (92)	147 (97)	<0.001
SBP (mm Hg)	126 ± 17	121 ± 15	122 ± 12	130 ± 18	134.5 ± 16	<0.001
DBP (mm Hg)	79 ± 9	77 ± 9.0	78 ± 8.4	80 ± 9.5	81.5 ± 8.4	<0.001
Heart rate (beats/min)	78 ± 31	81 ± 43	75.5 ± 12.3	77 ± 15	76.5 ± 11.6	0.437
BMI (kg/m^2^)	27 ± 3	26.4 ± 3.4	27.3 ± 3.8	27.4 ± 3.22	27.50 ± 3.3	0.029
Hypertension, *n* (%)	181 (29)	82 (27)	19 (40)	63 (47)	106 (70)	<0.001
Diabetes mellitus, *n* (%)	53 (8.4)	16 (5.3)	14 (10.3)	3 (6.3)	30 (19.6)	<0.001
Current smoking, *n* (%)	109 (17)	21 (7.0)	4 (8.5)	36 (27)	48 (32)	<0.001
Use of lipid lowering drugs, *n* (%)	132 (21)	45 (13.7)	7 (15)	21 (16)	37 (24.5)	<0.001
Use of antihypertensive drugs, *n* (%)	139 (22)	22 (7.3)	14 (30)	39 (29)	64 (42)	<0.001
CAC (MX-130)	123 ± 331	10.5 ± 31.4	498 ± 335	6.4 ± 14.6	335 ± 463	<0.001
CAC > 0, *n* (%)	336 (53)	94 (31)	47 (100)	51 (38)	151 (100)	<0.001
HDL (nmol/L)	1.2 ± 0.4	1.3 ± 0.4	1.3 ± 0.3	1.1 ± 0.3	1.1 ± 0.2	<0.001
Cholesterol (nmol/L)	2.2 ± 0.4	2.2 ± 0.4	2.2 ± 0.3	2.3 ± 0.3	2.2 ± 0.4	0.704
Triglyceride (nmol/L)	1.6 ± 0.9	1.5 ± 0.8	1.4 ± 1	1.8 ± 1	1.8 ± 0.9	<0.001
LDL (nmol/L)	3.3 ± 1.3	3.4 ± 1.7	3.2 ± 0.7	3.3 ± 0.7	3.3 ± 0.8	0.781
GFR (mL/min/1.73 m^2^)	101 ± 20	99 ± 21	104 ± 20	102.5 ± 21	103.5 ± 16	0.655
Glucose (nmol/L)	5.5 ± 1.3	5.2 ± 0.8	5.5 ± 1.3	5.7 ± 1.5	6.1 ± 1.7	<0.001
Calcium (nmol/L)	2.4 ± 0.1	2.4 ± 0.1	2.4 ± 0.1	2.4 ± 0.2	2.4 ± 0.1	0.380

*PCE, pooled cohort equations; MESA, multiethnic study of atherosclerosis; SBP, systolic blood pressure; DBP, diastolic blood pressure; BMI, body mass index; CAC, coronary artery calcium.*

### Event Rate

During the follow-up period of up to 11 years (mean 6.5 ± 3.3), 52 (8.2%) subjects experienced a first CV event, of which 28 had a coronary event and 24 had a CV event (Table 2). The highest CV event rate was noted in those with an elevated CV risk (i.e., >7.5%) as defined by either PCE or MESA-C scores: 12% (35/285) and 18.7% (37/198), respectively (Table 2). Conversely, the lowest CV event rate was noted in those with reduced CV risks (i.e., <5%) as defined by either PCE or MESA-C scores: 11/229 (4.8%) and 11/351 (3.1%), respectively (Table 2).

**TABLE 2:** Paired comparisons of MESA and PCE categories for cardiovascular disease, including stroke.

		MESA score			Total
		<5%	5–7.5%	>7.5%	
*PCE score*	<5%	↔184	↑20	↑25	229
	CV events, *n* (%)	7 (3.8)	1 (5.0)	3 (12.0)	11 (4.8%)
	5–7.5%	↓77	↔19	↑22	118
	CV events, *n (%)*	1 (1.3)	0	5 (22.7)	6 (5.1)
	>7.5%	↓90	↓44	↔151	285
	CV events, *n* (%)	3 (3.3)	3 (6.8)	29 (19.2)	35 (12.2%)
Total		351	83	198	632
	Total CV events, *n* (%)	11 (3.1)	4 (4.8)	37 (18.6)	52 (8.2)

*PCE, pooled cohort equations; MESA, multiethnic study of atherosclerosis; CV, cardiovascular.*

*↔Unchanged risk stratification.*

*↑Higher MESA-C score compared to PCE.*

*↓Lower MESA-C score compared to PCE.*

The highest CV event rate was observed in those who had a risk ≥ 7.5% according to the MESA-C score regardless of the PCE risk: 19.2% (29/151) when PCE risk was ≥ 7.5 and 17% (8/47) in those with a PCE risk of < 7.5% (Table 2). A MESA-C risk score of < 7.5% was associated with a low event rate regardless of PCE risk groups: 3% (9/300) in those with PCE risks of < 7.5 and 4.5% (6/134) for those with a PCE score of ≥ 7.5% (Table 2). A multivariate adjusted model compared patients in the lowest CV risk category by both scores, and the highest HR for a cardiac event was noted for those with a CV risk ≥ 7.5% by both scores: HR 9.1 (3.1–26.6) (Table 3).

**TABLE 3:** HR for cardiac events as well as total cardiovascular events according to the cardiovascular risk score classification.

	PCE score ≤ 7.5% & MESA-C score ≤ 7.5% *N* = 300	PCE score > 7.5% & MESA-C score ≤ 7.5% *N* = 134	PCE score ≤ 7.5% & MESA-C score > 7.5% *N* = 47	PCE score > 7.5% & MESA-C score > 7.5% *N* = 151
Cardiac event rate *n* (%)	7 (2.3)	4 (3)	8 (17)	25 (17)
Unadjusted HR	1	1.8 (0.5–6.6)	10.5 (3.4–32)	10.7 (4.1–28.1)
Multivariate adjusted* HR	1	1.6 (0.4–6)	7.8 (2.4–25.3)	9.1 (3.1–26.6)
CV event rate, *n* (%)	7 (2.3)	6 (4.5)	9 (19.1)	30 (20)
Unadjusted HR	1	2.7 (0.8–8.9)	11.9 (4–35.6)	12.9 (5–33.5)
Multivariate adjusted* HR	1	2.6 (0.8–8.9)	9.1 (2.9–28.6)	11.7 (4.1–33.2)

*Adjusted for hypertension, diabetes, smoking status, pulse, family history, lipid lowering and anti-hypertension drugs.

*PCE, pooled cohort equations; MESA, multiethnic study of atherosclerosis; SBP, systolic blood pressure; DBP, diastolic blood pressure; BMI, body mass index; CAC, coronary artery calcium.*

**FIGURE 1:**
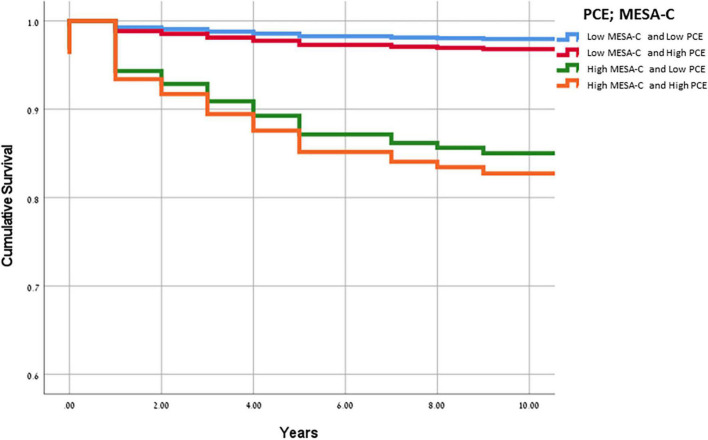
Kaplan-Meier curves for cumulative survival according to the cardiovascular risk score classification. Kaplan-Meier curves for cumulative survival according to the cardiovascular risk score classification. The blue line denotes low MESA-C and PCE risk categories (≤7.5% for both), the red line denotes low MESA-C risk (≤7.5%) and high PCE risk (>7.5%), the green line denotes high MESA-C risk (>7.5%) and low PCE risk (≤7.5%), and the orange line denotes high MESA-C and high PCE risk categories (>7.5% for both). PCE, pooled cohort equations; MESA, multiethnic study of atherosclerosis.

The HR for a cardiac event among those with PCE scores of ≥ 7.5% and MESA-C scores of ≤ 7.5% was 1.6 (0.4–6), while those with MESA-C scores of ≥ 7.5% and PCE scores of < 7.5% had an HR of 7.8 (2.4–25.3) (Table 3). Similar findings were demonstrated for CV events. Compared to patients in the lowest CV risk category by both scores, the highest HR for a CV event was noted for those with a CV risk ≥ 7.5%, by both scores: HR 11.72 (4.1–33.2) (Table 3). The HR for a CV event among those with PCE scores of ≥ 7.5% and MESA-C scores of < 7.5% was 2.6 (0.8–8.9), while those with MESA-C scores of ≥ 7.5% and PCE scores of < 7.5% had an HR of 9.1 (2.9–28.6) (Table 3). Accordingly, the cumulative survival for subjects in the various subgroups is presented in Figure 1. Subjects with low MESA-C scores had the highest survival rate regardless of the PCE risk, while those with high MESA-C risks had the lowest survival rate regardless of the PCE risk (Figure 1). Patients with low MESA-C scores but high PCE scores show lower survival compared to those with low MESA-C scores and low PCE scores, and those with high MESA-C scores and low PCE scores show higher survival compared to those with both scores being high (Figure 1). When survival is stratified by the MESA-C, there is no significant difference in survival between high and low PCE scores (*p* = 0.102 and *p* = 0.823 for low MESA-C scores and high MESA-C scores, respectively) (data not shown).

### Recategorization and Reallocation to Statin Treatment

**FIGURE 2:**
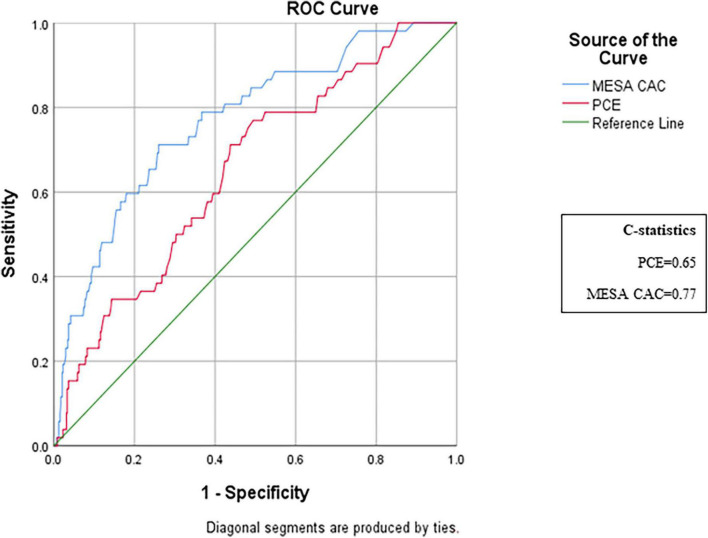
ROC analysis of the discriminative ability for the prediction of cardiovascular events by PCE and MESA risk scores. ROC curves comparing the predictive ability of MESA-C and PCE scores. The MESA-CAC curve is designated in blue, the PCE curve is in red, and the reference line is in green. ROC, receiver operating characteristic; PCE, pooled cohort equations; MESA, multiethnic study of atherosclerosis.

A total of 118 subjects had borderline PCE risk (5–7.5%), for whom a statin is not clearly indicated. Of them, 99 (84%) were recategorized according to their MESA-C score with 22 (22%) being up-categorized, thus becoming statin eligible, while 77 (78%) were down-categorized and thus statins were not indicated (Table 2). A total of 403 patients had a PCE risk score > 5%, and thus were mostly statin eligible. Of them, 167 (41%) had a MESA-C score of less than 5%, thus statins were actually not indicated (Table 2). Categorization of CV risk was concordant for both PCE and MESA-C scores in 354 subjects (Table 2). Of them, 203 (57%) had a CV risk < 7.5% by both scores and 151 (43%) had a CV risk ≥ 7.5% by both scores (Table 2). For 278 subjects (44%), allocation to a CV risk category was not concordant between the two risk scores. These participants were recategorized according to the MESA-C score, with 211 (76%) being recategorized to a lower CV risk category than previously defined by the PCE, and 67 (24%) subjects being recategorized to a higher CV risk category (Table 2). Of the 211 subjects with higher PCE scores (>5%) and lower MESA-C scores, 44 (21%) were reallocated to an intermediate CV risk category of 5–7.5%, while 167 (79%) were re-categorized to the lowest risk of < 5% (Table 2). An ROC analysis for the discriminative ability for prediction of CV events by both scores showed a C-statistics of 0.653 for the PCE and 0.77 for the MESA-C score (Figure 2).

### Zero Coronary Artery Calcium

**FIGURE 3:**
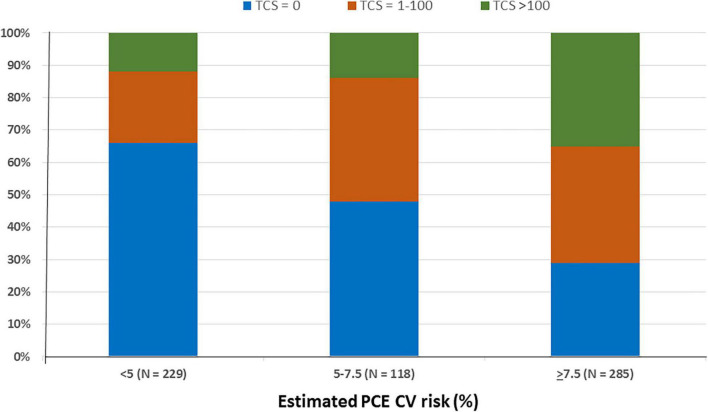
Coronary artery calcification according to the estimated PCE CV risk. The subjects (632) were divided according to their PCE CV risk and total calcium score. Bars represent the estimated PCE CV risk category. Colors represent the total calcium score. N denotes the absolute number of subjects according to their PCE CV risk category. PCE, pooled cohort equations; CV, cardiovascular; TCS, total calcium score.

Of all study participants, 296 (47%) had zero CAC (Table 1). Of them, 99% had a MESA-C risk of < 5% and thus statins were not indicated, while 140 subjects (47%) had PCE scores > 5% (Figure 3). Of the 285 subjects classified as having ≥ 7.5% by PCE, 83 (29%) had zero CAC, and of 403 subjects with an estimated PCE risk of > 5%, 140 (34%) had zero CAC (Figure 3).

## Discussion

In our cohort of asymptomatic adults, we demonstrated that the MESA-C risk score can improve allocation to statin treatment. The ability of the MESA-C score to improve our discriminative capacity is mainly due to the inclusion of the CAC score into the MESA algorithm. Interestingly, the CAC appears to be of importance when the MESA-C scores and PCE scores are non-concordant. This is of particular importance in light of the various professional society guidelines recommendations on cholesterol treatment which significantly increases the eligibility for statin therapy (3, 4, 6). Our data show that when the MESA-C score is applied, almost half of study subjects are recategorized to a different CV risk class, and the majority of the reallocations are to a lower CV risk category than previously defined by the PCE. Consequently, statin eligibility also changed. Approximately 85% of subjects with borderline PCE risk (i.e., 5–7.5%), who are questionably eligible for statins, were recategorized according to their MESA-C score. The majority of these participants were down-categorized with statin treatment no longer be indicated. In addition, almost two-thirds of our study population had a PCE risk score > 5% (i.e., candidate for statin therapy). However, almost half of them had a MESA-C score of < 5%, and thus statins were eventually not indicated. Our findings concur with previously published data from a larger study by Nasir et al. (37).

In addition, we also present data regarding survival calculation by these scores. Our Kaplan–Meier’s curves for survival according to the MESA-C and PCE risk scores show that compared to both scores being low, a high PCE score predicts low survival, and compared to both scores being high, a low PCE score predicts better survival. These data suggest that both scores are essential for more complete survival analysis.

CAC is a well-established surrogate marker for the total burden of coronary artery atherosclerosis. Its major advantage lies in that it measures sub-clinical CHD that reflects the entire genetic, metabolic, and acquired risk factors that determine the coronary atherosclerotic burden and progression. This explains the incremental prognostic value of CAC for the prediction of CV events and mortality beyond traditional risk factors (7, 8, 10–14, 22, 32, 37–41). The MESA-C study was a population-based study with a multiethnic composition and availability of a 10-year follow-up for CHD event incidents (38). The MESA-C risk score is the only risk score that optimizes 10-year CHD risk prediction by including CAC in addition to traditional risk factors (36), allowing it to be a potent adjunct tool in risk-based treatment decisions in the clinical practice (42, 43). Polonsky et al. (11) demonstrated that adding CAC to the MESA risk prediction model significantly alters the risk classification that was previously based solely on traditional risk factors. The main clinical contribution of CAC is its impact on statin therapy for primary prevention. Mitchell et al. (24) followed patients without baseline CVD for a median of 9.4 years and found that statin therapy was associated with a reduced risk of CV events in patients with measurable CAC, but not in patients with zero CAC and that the effect of statin use on CV events was significantly related to the severity of CAC. Most recently, a large retrospective cohort study of 53,487 patients with a mean follow-up of 12 years supports the use of CAC for further risk assessment in the borderline and intermediate risk groups. Furthermore, this study has shown that even in select subjects at low or high estimated risk, CAC modestly improves risk discrimination (34). Our findings agree with the aforementioned studies. However, we provide additional data regarding the discriminative capacity of the MESA-C score in a different patient population, which further contributes to the clinical validation of the MESA-C score. Moreover, our data emphasize the significance of risk recategorization and reallocation to an appropriate CV risk group, thereby obtaining a more informed decision regarding statin treatment.

The absence of CAC indicates an excellent CV prognosis and has a high negative predictive value for CV event occurrences (23, 25–29, 44). We found that almost one third of those who were classified by the PCE to the higher risk category of ≥ 7.5% had zero CAC, and consequently were recategorized by the MESA-C risk calculator to the lowest CV risk category. Nasir et al. evaluated the implications of zero CAC on risk score reclassification (37) and demonstrated that the absence of CAC reclassifies 44% of those who were defined as eligible for statins by the PCE, as not eligible. Similarly, we found that almost half of those who were classified by the PCE as eligible for statins had zero CAC and were therefore not eligible for statins by the MESA-C categorization. Furthermore, we found that zero CAC resulted in a MESA-C risk of < 5% in 99% of the cases and was associated with a very low event rate. We showed that when CAC is included in the MESA score, the discrimination capacity of MESA-C, as reflected by the ROC analysis, increases from a C-statistics of 0.653 to 0.770. Our findings concur with those suggested by Budoff et al. (10), who found that the 10-year event rates in those with zero CAC were almost exclusively below 5%, while those with CAC ≥ that the 10-year event rates in those with zero CAC were almost exs on cholesterol treatment (1) suggest statin treatment for all adults 40–75 years with CAC ≥ 100.

Our study has several limitations. First, this is a retrospective analysis with all of its inherent biases. Second, the information regarding statin treatment was available only at baseline but the use or initiation of statin during follow-up is lacking. Nevertheless, cholesterol levels at baseline were not statistically different between the study groups and they are also included in the MESA-C score algorithm. Third, our study is limited to subjects within the age range of the MESA-C study (45–80 years). Therefore, our findings cannot be applied to younger than 45 years. However, it has been previously shown that CAC is a risk modifier in the younger population as well (45), particularly, in those with a family history of CHD (46). Fourth, the MESA-C risk algorithm was designed to estimate only the cardiac risk, while the PCE also includes a composite vascular risk assessment. Notably, it has been previously demonstrated that CAC is an independent predictor of CVA events in the asymptomatic MESA cohort and improves the discrimination afforded by current stroke risk factors (31). Accordingly, we were able to demonstrate the relevance of the MESA-C risk categorization for overall CV risk, including CVA events. Finally, we excluded one hundred and thirteen subjects from our initial cohort due to incomplete data, thus our final cohort may also be at risk for selection bias.

## Conclusion

In conclusion, our study provides further clinical validation for CAC and emphasizes the advantage of the MESA-C risk score as a discriminative tool for CV risk stratification and thus eligibility for statin use. Based on our data and current literature, the MESA-C score may serve as an important adjunct tool in our armamentarium for patient-physician decision-making and primary CV prevention.

## Data Availability Statement

The original contributions presented in the study are included in the article/supplementary material, further inquiries can be directed to the corresponding author.

## Ethics Statement

The studies involving human participants were reviewed and approved by the Chaim Sheba Medical Center Helsinki Committee. Written informed consent for participation was not required for this study in accordance with the national legislation and the institutional requirements.

## Author Contributions

GS, JS, SS, and EG performed the material preparation, data collection, and analysis. GS and JS wrote the first draft of the manuscript. NK-M performed the statistical analysis. All authors commented on previous versions of the manuscript, contributed to the study conception and design, and read, and approved the final manuscript.

## Conflict of Interest

The authors declare that the research was conducted in the absence of any commercial or financial relationships that could be construed as a potential conflict of interest.

## Publisher’s Note

All claims expressed in this article are solely those of the authors and do not necessarily represent those of their affiliated organizations, or those of the publisher, the editors and the reviewers. Any product that may be evaluated in this article, or claim that may be made by its manufacturer, is not guaranteed or endorsed by the publisher.
